# Integrating Research, Policy and Practice to Address Antimicrobial Resistance in Aotearoa New Zealand: The NZAMRNet Initiative

**DOI:** 10.1002/snz2.70079

**Published:** 2026-08-19

**Authors:** Ghader Bashiri, Juliet A. Gerrard

**Affiliations:** ^1^ School of Biological Sciences The University of Auckland Private Bag New Zealand

**Keywords:** AMR impact, AMR response, antimicrobial resistance

## Abstract

Antimicrobial resistance (AMR) is an escalating global challenge with significant implications for human health, agriculture and environmental sustainability in Aotearoa New Zealand. As for many complex ‘wicked problems’, addressing AMR requires coordinated, transdisciplinary approaches that integrate research, policy and practice; however, current efforts remain fragmented, limiting national impact and readiness to respond to this complex threat. Here, we describe the establishment of the Aotearoa New Zealand Antimicrobial Resistance Network (NZAMRNet), a national collaborative initiative to strengthen coordination across the AMR landscape. NZAMRNet provides an enabling platform to connect researchers, clinicians, industry and policymakers, supporting aligned research activity, knowledge exchange and evidence‐informed decision‐making. We outline the network's strategic focus and its role in enhancing collaboration, building capability and improving translation into practice and policy. NZAMRNet strengthens national coordination and positions Aotearoa New Zealand to lead integrated regional responses to AMR. NZAMRNet represents an important step toward a more coherent, adaptive and impactful response to AMR.

## 1 Antimicrobial Resistance in Aotearoa New Zealand

Antimicrobial resistance (AMR), whereby micro‐organisms evolve to withstand antimicrobial agents, is recognised as one of the most pressing global public health challenges, impacting human and animal health, food production systems and environmental sustainability (Naghavi et al. 2024; Global Antibiotic Resistance Surveillance Report 2025). These challenges are particularly significant for Aotearoa New Zealand given the close interconnections between human health, primary industries and environmental stewardship. Yet, Aotearoa New Zealand lacks integrated, quantitative estimates of the national AMR burden (The Office of the Prime Minister's Chief Science Advisor 2022). Current estimates indicate that in 2022, resistant infections imposed direct costs exceeding $130 million and approximately 278 deaths directly attributable to AMR, with a further 1300 deaths associated with resistant infections nationwide (McDonnell et al. 2024). To place this in context, the annual road toll in Aotearoa New Zealand is typically between 290 and 340 deaths (Ministry of Transport 2026), highlighting that AMR already represents a comparable, if not greater, mortality burden. In addition, AMR poses potential risks to primary industries such as dairy and horticulture (Ministry for Primary Industries 2025), although its implications for exports and food security remain poorly characterised. The breadth of these impacts underscores the need for coordinated national leadership across healthcare, agriculture, food production and environmental sectors.

Aotearoa New Zealand has taken important steps to address AMR at a policy level. A five‐year Antimicrobial Resistance Action Plan was introduced in 2017 (Ministry of Health 2025) following the World Health Organization's global strategy. Subsequent initiatives, including the *Kotahitanga* report from the Office of the Prime Minister's Chief Science Advisor, have emphasised the need for a unified national response (The Office of the Prime Minister's Chief Science Advisor 2022). In addition, a new National AMR Strategy is currently under development to guide future response. These frameworks have largely prioritised awareness, prevention, surveillance and stewardship. While essential, the continued emergence and spread of AMR suggest that these measures alone are insufficient to address the evolving nature of the problem and highlight the need for greater investment in research, innovation and evidence generation. Without sustained investment in the discovery and development of new infection prevention tools, diagnostics and therapeutics and policy interventions, our responses to AMR are at risk of becoming reactive rather than anticipatory, reducing their long‐term effectiveness and leaving us unprepared for the introduction of an untreatable pathogen to New Zealand.

Aotearoa New Zealand has a strong and diverse AMR research community spanning universities, Public Research Organisations, clinical settings and government agencies. However, these efforts remain largely isolated within sectors and institutions, limiting their collective impact. Connections between research, clinical practice and policy are often indirect, and engagement with industry is variable, slowing the translation of knowledge into practical solutions. These challenges highlight a critical gap between national capability and coordinated national action, underscoring the need for mechanisms that can integrate expertise, align priorities and enhance collaboration across the AMR landscape. Internationally, organisations are emerging to bridge these gaps and connect internationally (MTPConnect; Duddy and Harker 2026).

## 2 NZAMRNet: A National Collaborative Initiative

In response to these challenges, the Aotearoa New Zealand Antimicrobial Resistance Network (NZAMRNet) has been established as a national collaborative initiative. NZAMRNet is designed as an enabling framework that provides a unifying platform to connect stakeholders across research, clinical practice, industry and policy. The network spans the full AMR continuum, from fundamental research on microbial biology and resistance mechanisms to clinical practice, public health and policy development, thereby bridging traditional boundaries that currently limit impact. The core role of NZAMRNet is to strengthen connections across the AMR ecosystem and support coordinated approaches to research and implementation. By bringing together researchers across sectors, clinicians, industry partners, policymakers and community stakeholders, NZAMRNet facilitates alignment of priorities and supports the integration of scientific, environmental, clinical, veterinary and policy perspectives. The network also recognises the value of patient and community engagement, particularly from groups disproportionately affected by resistant infections or whose healthcare depends on effective antimicrobials, including cancer patients, transplant recipients and those undergoing major surgery. NZAMRNet strives for real‐world impact by engaging with these groups to ensure research priorities, policy discussions and collaborative activities remain aligned with community needs.

NZAMRNet undertakes activities across several key areas: (1) coordinating research efforts nationally, reducing duplication and improving alignment with strategic priorities; (2) facilitating knowledge exchange through workshops, symposia and collaborative platforms that support interdisciplinary engagement; and (3) promoting innovation and translation by connecting academic research with end users, including industry and healthcare providers, and supporting engagement with policy stakeholders to inform evidence‐based decision‐making.

An important principle underpinning NZAMRNet is the inclusion of Māori and Pacific perspectives. The network seeks to foster partnerships and approaches that align with Te Ao Māori values, supporting equitable participation and ensuring that AMR research and policy are relevant and responsive to communities across Aotearoa New Zealand (The Office of the Prime Minister's Chief Science Advisor 2022). As part of our developing governance structure, we are currently establishing a Māori working group, which spans early‐career researchers to clinicians, to facilitate and foster these partnerships.

## 3 Opportunities for National and Regional Impact

By strengthening coordination at a national level, NZAMRNet creates opportunities to enhance Aotearoa New Zealand's response to AMR and maximise the impact of existing capabilities. By aligning research activity with policy priorities and health system needs, the network supports more effective use of research investment and facilitates the development of collaborative initiatives that can achieve impact at scale. This, in turn, improves the translation of research into prevention, diagnostics, therapeutics and stewardship strategies.

The establishment of NZAMRNet also has important regional implications, particularly across Australasia and the Pacific (Yam et al. 2019). AMR is inherently transboundary: Resistant organisms, genes and resistance move across borders through human mobility, trade and environmental pathways (Graham et al. 2019; Davido et al. 2026). Across the Western Pacific and broader Indo‐Pacific region, surveillance capacity, access to diagnostics and health system resources remain inconsistent and inequitable (Giri and Lewycka 2026). Limited data in some settings can obscure the true scale of AMR, while resource constraints increase vulnerability to its emergence and spread (Gandra et al. 2020). Pacific Island countries are particularly affected, with growing AMR burdens expected to have substantial health and economic impacts in the coming decades (Piukala and Boehme 2026).

In this context, NZAMRNet seeks to deepen engagement with Australian and Pacific partners to align existing regional initiatives, offer opportunities to coordinate research priorities, share infrastructure and data and co‐develop responses to shared challenges. By embedding national efforts within this broader regional framework, NZAMRNet can significantly build domestic capability while also enhancing regional health security.

The relevance of regional coordination was reinforced at the 2026 AMR Summit in Australia, which emphasised the need to move beyond fragmented efforts toward implementation‐focused, cross‐sector collaboration. Key priorities included improving data sharing, aligning investment and developing integrated approaches that bridge research, policy and practice (AMR Summit 2026). These priorities closely align with the objectives of NZAMRNet and highlight the opportunity for Aotearoa New Zealand to contribute to, and benefit from, a connected regional AMR ecosystem.

## 4 Anticipated Outcomes, Challenges and Future Directions

NZAMRNet is expected to deliver system‐level benefits across the AMR landscape. Central to this is the development of a more coherent and coordinated national research agenda, supported by enhanced collaboration and improved access to multi‐institutional funding. By linking expertise across disciplines and sectors, the network strengthens the foundations for impactful and scalable research.

The network also supports translation into policy and practice by connecting researchers with clinicians, veterinarians, industry and policymakers; transdisciplinary linkages that are often challenging for small research teams to establish when working in isolation. This facilitates the development of evidence‐based guidelines and informs national strategies, streamlines workloads while reducing duplication of effort and ensures that scientific advances are more readily and rapidly incorporated into decision‐making.

Achieving these outcomes will not be without challenges. While AMR research and management in Aotearoa New Zealand already benefit from a range of established relationships and sector‐specific initiatives, coordination across the broader AMR landscape remains difficult. Researchers, clinicians, industry, government agencies and policymakers often operate with different priorities, objectives and timeframes, creating barriers to sustained collaboration. There is also a risk that network activities may remain largely informational unless clear opportunities are created for collaboration, alignment of priorities and translation into practice. NZAMRNet seeks to address these challenges by providing a dedicated national platform that connects stakeholders across sectors and disciplines, helping to transform existing relationships into a more coordinated and strategically aligned AMR community.

NZAMRNet's future effectiveness will depend on its ability to sustain and expand this integrated approach. This includes deepening partnerships across sectors, strengthening engagement with Māori and Pacific communities and building connections with regional and international initiatives. Continued support from institutions, funders and policymakers will be essential to maintain momentum and ensure long‐term impact.

## 5 Concluding Remarks

AMR represents a complex and evolving challenge that spans human health, primary industries and the environment in Aotearoa New Zealand. While the country has strong capabilities across these domains, fragmented efforts have limited their collective impact. NZAMRNet provides a timely mechanism to address this gap by strengthening collaboration and alignment across the AMR landscape. By supporting a more coordinated and forward‐looking approach, the network has the potential to enhance Aotearoa New Zealand's capacity to anticipate and respond to AMR challenges. Sustained commitment to this integrated model will be critical to ensuring a resilient and effective national response in the years ahead.

## Funding

The authors have nothing to report.

## Conflicts of Interest

The authors declare no conflicts of interest.

## Data Availability

Data sharing is not applicable to this article as no datasets were generated or analysed during the current study.
